# An electrochemically synthesized molecularly imprinted polymer for highly selective detection of breast cancer biomarker CA 15-3: a promising point-of-care biosensor†

**DOI:** 10.1039/d4ra02051k

**Published:** 2024-05-13

**Authors:** Daniela Oliveira, Yonny Romaguera Barcelay, Felismina T. C. Moreira

**Affiliations:** a CIETI – LabRISE-School of Engineering, Polytechnic of Porto R. Dr António Bernardino de Almeida, 431 4249-015 Porto Portugal; b CEMMPRE, Department of Chemical Engineering, University of Coimbra Rua Sílvio Lima – Pólo II 3030-790 Coimbra Portugal; c BioMark@ISEP, School of Engineering of Polytechnique School of Porto Porto Portugal ftm@isep.ipp.pt; d LABBELS/CEB, Centre of Biological Engineering, University of Minho Braga Portugal

## Abstract

In this study, a molecularly imprinted polymer film (MIP) was prepared on the surface of a disposable carbon screen-printed electrode (C-SPE) using (3-acrylamidopropyl)trimethylammonium chloride (AMPTMA) as a functional monomer and the cancer biomarker carbohydrate antigen 15-3 (CA 15-3) as a template. The MIP was synthesized by *in situ* electropolymerization (ELP) of the AMPTMA monomer in the presence of the CA 15-3 protein on the C-SPE surface. The target was subsequently removed from the polymer matrix by the action of proteinase K, resulting in imprinted cavities with a high affinity for CA 15-3. Electrochemical techniques such as cyclic voltammetry (CV) and electrochemical impedance spectroscopy (EIS) were used to characterize the different phases of the sensor assembly. Chemical and morphological analysis was performed using RAMAN and scanning electron microscopy (SEM). CA 15-3 was successfully detected in a wide working range from 0.001 U mL^−1^ to 100 U mL^−1^ with a correlation coefficient (*R*^2^) of 0.994 in 20 min. The MIP sensor showed minimal interference with other cancer proteins (CEA and CA 125). Overall, the developed device provides a rapid, sensitive, and cost-effective response in the detection of CA 15-3. Importantly, this comprehensive approach appears suitable for point-of-care (PoC) use, particularly in a clinical context.

## Abstract 2

Here, a molecularly imprinted polymer film (MIP) was prepared on the surface of a disposable carbon screen-printed electrode (C-SPE) using (3-acrylamidopropyl)trimethylammonium chloride (AMPTMA) as a functional monomer and the cancer biomarker carbohydrate antigen 15-3 (CA 15-3) as a template.

## Graphical abstract

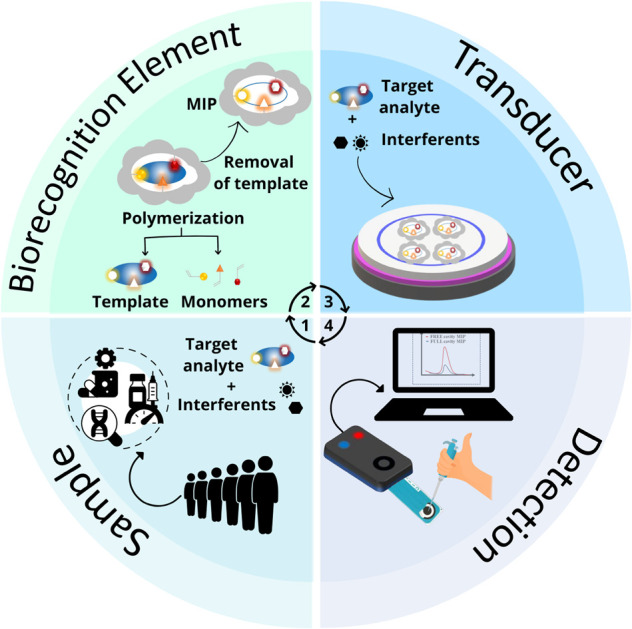

## 1. Introduction

Glycoproteins play crucial roles in fundamental biological processes such as molecular recognition, cell signaling, the immune response, and the regulation of cell development. Therefore, the identification of glycoprotein levels has intensified since the expression of these substances can provide direct information on the evolution of pathological conditions and the physiological state.^1,2^ CA 15-3, a glycoprotein of approximately 400 kDa like mucin 1 (MUC1), is secreted by breast cancer cells and is the most commonly used serum biomarker for monitoring breast cancer.^3^

Glycoproteins normally occur in an organism either as soluble or membrane-bound molecules. These glycoproteins can differ according to the type, length, and linkage of the carbohydrate components and even the degree of saturation of the potential glycosylation sites on the protein itself.^4^ Divergent glycolysis is associated with differential expression of enzymes such as glycosyltransferases and glycosidases. The abnormal expression of these enzymes leads to cancer cells producing glycoproteins with specific, cancer-associated deviations in glycan structures.^5^ Some of these glycan structures and glycoproteins are known tumour markers, such as CA 15-3, which is used as a breast cancer marker.^6,7^

Thus, its primary applications cover post-operative surveillance, consideration of the continuation of a specific treatment, the possibility of discontinuing that therapeutic protocol, as well as the evaluation of transitioning to viable alternatives.^8,9^ Healthy individuals typically exhibit CA 15-3 levels below 30 U mL^−1^, in contrast to patients who, after surgery, show higher concentrations (>30 U mL^−1^). On such a basis, much attention has been devoted to developing procedures to detect the presence of CA 15-3 protein at very low concentrations in a physiological environment and biological fluids.^8^

Currently, detection of the CA 15-3 biomarker is often carried out using immunoassays that employ various signal transduction approaches, such as ELISA,^10,11^ flow fluorescence,^12^ chemiluminescence,^13,14^ electrochemiluminescence,^15,16^ and electrochemistry.^17,18^ Although these biosensors are highly selective due to the use of corresponding natural biomolecules such as enzymes, antibodies, aptamers, and lectins, they often present problems of high cost, tendency to denature, and low abundance. In addition, the procedures for these methods are often time-consuming and expensive.^19^ Given these limitations, there is growing interested in approaches that aim to mimic natural recognition systems using synthetic analogues of molecular imprinting polymers (MIPs).^20,21^ Plastic antibodies relying on MIPs are a cost-effective technology compared to natural recognition elements because they are easier to produce than natural antibodies as they are synthesized by chemical processes and avoid the complexity of biological systems. They are stable under different conditions, which lowers storage costs, and unlike natural antibodies, they are reusable, which lowers overall costs. In addition, MIPs can be customized for specific target molecules, which increases detection efficiency and cost-effectiveness in various applications.^20,21^

Overall, the adoption of the MIP strategy allows for the creation of more robust and effective biosensors, contributing to the overall improvement of CA 15-3 detection techniques.^22,23^

MIP synthesis entails polymerization utilizing functional monomers, cross-linkers, and initiator molecules in the presence of a template molecule. After polymerization, the template is removed from the polymer matrix, creating a cavity that precisely matches the size and shape of the target molecule. Consequently, this cavity exhibits a molecular memory that allows it to rebind to the template selectively and effectively with high affinity.^24–27^ As a result, MIPs facilitate the specific recognition of target molecules in their surrounding environment. Several biosensors utilizing MIPs have been documented in the literature for the detection of the CA 15-3 protein.^28–32^

Generally, the design of MIPS-based biosensors is based on radical or electropolymerization (ELP) techniques.^33,34^ The key benefits of this technique include the ability to regulate the polymer's growth rate through careful selection of ELP parameters, the thickness of the film by managing the current during deposition, and the film's morphology by choosing an appropriate solvent and support electrolyte.^35,36^ Hence, ELP is typically conducted on a conductive support material. To this end, any monomer acquiring an oxidation potential and the capability for oxidation in the solution can be employed. Among the most prevalent are pyrrole, aniline, methylene blue, 3,4-ethylenedioxythiophene (EDOT), aminophenol, dopamine, and many others^33,36^ for imprinting proteins.^28–31^

In this study, we investigated the use of quaternary ammonium cation as a functional monomer in the design of imprinted polymers for CA 15-3, an important model compound for chemically charged and highly water-soluble compounds. (3-acrylamidopropyl)trimethylammonium chloride (AMPTMA) was chosen for this purpose. Several studies in the literature describe the use of AMPTMA to imprinting different targets. The author Zarejousheghani M. *et. al.*,^37^ described a MIP sensor, specifically employing quaternary ammonium cations as functional monomers, to design efficient and selective sorbents for glyphosate, with promising results for water-soluble and polar targets in sensor applications. Additionally, Xiong H. *et. al.*,^38^ reported intelligent MIPs utilizing an *N*-isopropyl acrylamide/AMPTMA binary deep eutectic solvents (DESs) system, showcasing enhanced adsorption capability, eco-friendly thermo-regulated elution, and simple magnetic separation for selective extraction of rhein from Cassiae semen samples, demonstrating potential applications in complex sample analyses. Liu Z. and his co-workers^39^ have developed magnetic MIPs exclusively utilizing deep eutectic solvents that demonstrated exceptional selectivity for bovine hemoglobin, with a high adsorption capacity of 229.54 mg g^−1^, an imprinting factor of 21.89, and superior performance compared to traditional polymers, highlighting its potential for selective recognition in complex samples. Another study that also uses the AMPTMA monomer was carried out by Pluhar B. *et. al*.,^40^ it consists of the development of submicron-sized surface-imprinted polymer particles with high affinity (dissociation constant of 7.94 μM) and rapid equilibrium binding (1 min incubation), demonstrating specific selectivity for pepsin over other proteins, such as bovine serum albumin and β-lactoglobulin, and highlighting the influence of ionic interactions on achieved selectivity through competitive binding studies with α1-acid glycoprotein. As far as we know this is the first research work described in the literature integrating AMPTMA and ELP technique for the selective recognition of the CA 15-3 protein.

In this study, an MIP sensor was developed for the analysis of the CA 15-3 protein. The MIP was produced by combining CA 15-3 with a quaternary ammonium salt monomer, (AMPTMA), facilitating their interaction. Subsequently, the mixture was electropolymerized onto the surface of a screen-printed carbon electrode (C-SPE). Following protein extraction, cavities imprinted in the polymer matrix were formed, enabling the subsequent rebinding of the target molecule. The sensor's response to CA 15-3 was measured using a redox probe responsible for the electrochemical signal. This straightforward approach allowed for the selective and sensitive detection of CA 15-3 in serum, achieving a lower limit of linear response of 1.0 mU mL^−1^. Therefore, the proposed MIP sensor offers a non-destructive, rapid, practical, and quantitative testing method, holding promising applications in the realm of clinical diagnosis and prognosis.

## 2. Experimental section

### 2.1. Instruments

The electrochemical measurements were made in a potentiostat/galvanostat from Metrohm Autolab equipped with an FRA module and controlled by Nova 2.1.6 software. The C-SPEs (DRP-110, DropSens) contained a carbon working electrode (4 mm), a carbon counter electrode, and a silver pseudo-reference electrode, having electrical contacts made of silver. The switch box interfacing these C-SPEs and the potentiostat were obtained from DropSens.

Scanning Electron Microscopy (SEM) analysis was carried out at a high vacuum, 15 kV, and at a magnification of 25 to 100-fold. Images were acquired of the bare C-SPE, MIP, and NIP materials at various locations on each sample.

Raman spectra of the dried drops of supernatant were collected using a commercial Renishaw inVia microscope (Gloucestershire, UK) equipped with a 532 nm excitation laser and a 100× objective (laser spot size ≈ 1 μm^2^). The power on the sample was approximately 5 mW and the instrument was calibrated using the 520 cm^−1^ line of a silicon wafer. Images were acquired of the bare C-SPE, MIP, and NIP materials at various locations on each sample.

### 2.2. Reagents and solutions

All chemicals were of analytical grade and water was ultrapure Milli-Q laboratory grade. Potassium hexacyano trihydrate (K_4_Fe(CN)_6_·3H_2_O), potassium hexacyano dihydrate (K_3_Fe(CN)_6_), sodium phosphate dibasic dihydrate (Na_2_HPO_4_·2H_2_O) and sodium dihydrogen phosphate dihydrate (NaH_2_PO_4_·2H_2_O) were purchased from Riedel de Haën; fetal bovine serum (FBS) was purchased from Alfa Aesar; sulphuric acid (H_2_SO_4_) was obtained from BDH; urea was from Fagron; proteinase K and (3-acrylamidopropyl)trimethylammonium chloride (solution 75 wt% in H_2_O) (AMPTMA) was acquired from Sigma-Aldrich; carcinoembryonic antigen (CEA) was obtained from EastCostBio; Cancer Antigen 125 (CA-125) was from Hytest; CA 15-3 from host human (reference MBS536585) was purchased from MyBioSource.

For the calibration curves, CA 15-3 standard solutions ranging from 0.001 U mL^−1^ and 100 U mL^−1^ were used, prepared in PB buffer (pH 5.8). Each solution was incubated for 20 min at the electrode surface. Selectivity studies were conducted by competitive assay in which CA 15-3 (30 U mL^−1^) were mixed with CEA (2.5 ng mL^−1^), CA 125 (35 U mL^−1^), and urea (0.2 mg mL^−1^). All these solutions were prepared in PB buffer, at pH 5.8, in triplicate.

### 2.3. Electrochemical procedures and sample preparation

The electrochemical assays were conducted indirectly using 5.0 mmol L^−1^ K_3_[Fe(CN)_6_] and 5.0 mmol L^−1^ K_4_[Fe(CN)_6_] as a redox probe prepared in phosphate buffer (PB) and electrochemical impedance spectroscopy techniques (EIS) and cyclic voltammetry (CV) were used to characterize the sensors in different steps of the biosensor assembly.

Square wave voltammetry (SWV) assays were also conducted in triplicate with the same redox couple [Fe(CN)_6_]^3−/4−^ operated under the following conditions: step potential of 5 mV, pulse amplitude of 10 mV, and frequency of 5 Hz.

### 2.4. Assembly of the plastic antibody on C-SPE

**Fig. 1:**
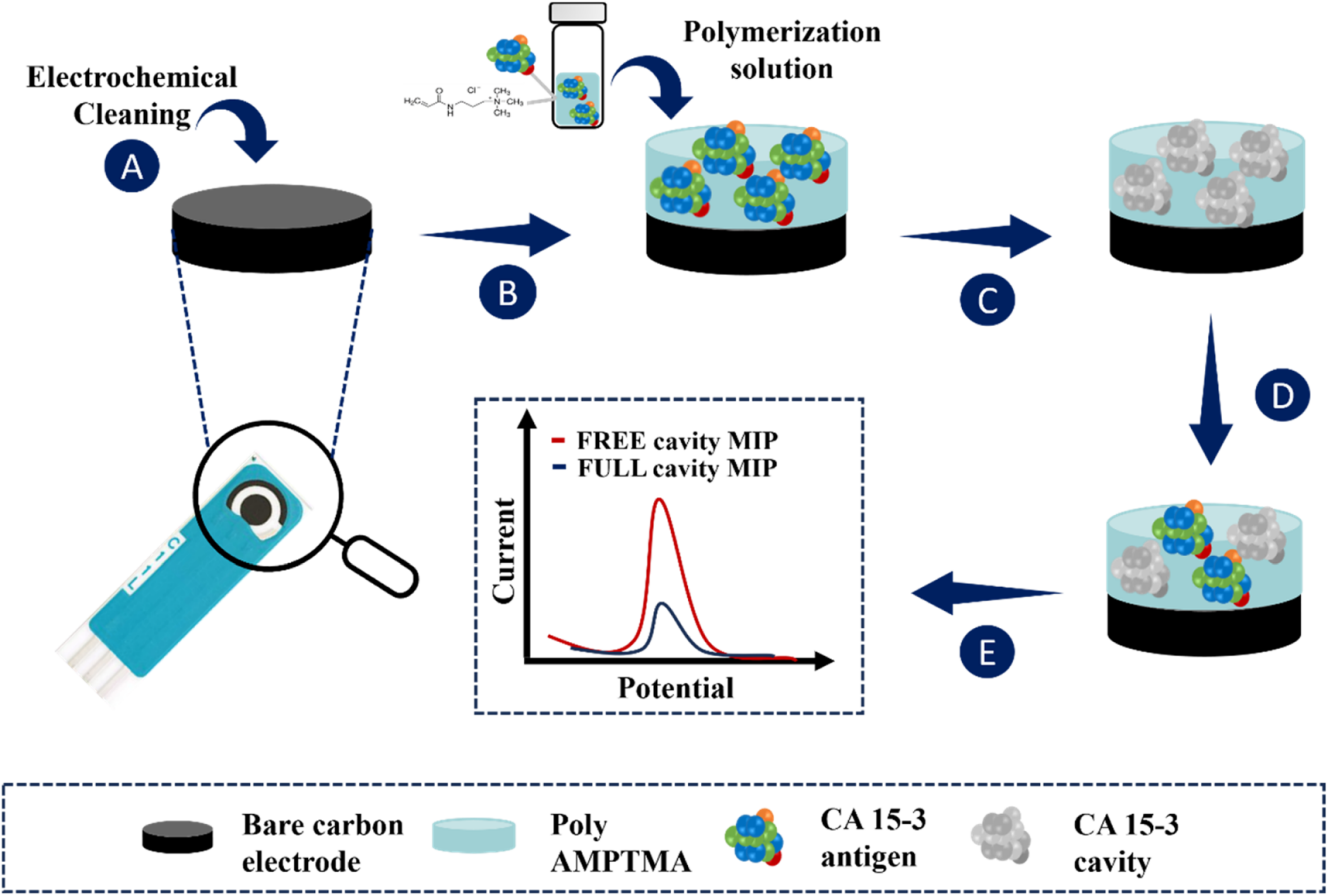
Schematic representation of a MIP for the detection of the CA 15-3 protein: (A) work electrode pre-treatment; (B) ELP of a solution containing CA 15-3 protein and monomer (AMPTMA); (C) CA 15-3 protein removal from polymer matrix; (D) template binding on the MIP surface; (E) analytical performance of the sensor.

The application of the MIP film to the working electrodes of the C-SPEs is shown in Fig. 1. First, the C-SPEs (Fig. 1A) were subjected to electrochemical cleaning using 0.5 mol L^−1^ H_2_SO_4_. The CV scans covered a potential range from −0.2 to +1.5 V at a scan rate of 0.05 V s^−1^ for 10 cycles. This step is crucial as it facilitates activation and uniformity of the working range across different electrodes.^41,42^ Subsequently, 5 μL of the polymerization solution, consisting of 50 mmol L^−1^ AMPTMA monomer and 100 U mL^−1^ CA 15-3 (as target molecule), prepared in PB buffer pH 5.8, was applied to the surface of the pretreated working electrode (WE) (Fig. 1B). ELP was performed by CV in a potential range of −0.4 to +0.7 V with a potential scan rate of 0.05 V s^−1^ for ten consecutive cycles. Finally, the target molecule was removed by incubating 5 μL of a solution containing 500 μg mL^−1^ proteinase K on the WE overnight at 4 °C (Fig. 1C).

Non-imprinted polymers (NIPs) are generated following the same procedure as imprinted polymers, but without including the target molecule in the ELP solution. These NIPs act as references in protein recognition experiments. They are crucial as negative controls in imprinted polymer research, allowing comparison with MIPs to evaluate selectivity. Additionally, NIPs are useful for investigating non-specific interactions between polymers and other molecules, providing a reference point to distinguish specific and non-specific effects, thereby contributing to understanding the properties and performance of imprinted polymers.

## 3. Results and discussion

### 3.1. Fabrication of the biosensor

In this work, we developed an electrochemical biosensor that uses a molecularly imprinted polymer to detect the tumor marker CA 15-3 in PoC tests. The ELP of AMPTMA is possible due to the presence of reactive functional groups in the monomer and the application of an electrical potential.^43,44^

ELP is a technique in which an electric current is used to initiate and drive the polymerization of monomers, resulting in the formation of a polymer film on an electrode surface. Here are some key factors that contribute to the ELP of AMPTMA: (i) reactivity of the double bond: AMPTMA contains an acrylamide group that has a reactive double bond (C=C). This double bond is susceptible to polymerization reactions, especially under the influence of an external electric field, and (ii) cationic nature: the presence of a trimethylammonium group in AMPTMA introduces a cationic charge. The cationic character increases the reactivity of the monomer and can facilitate the electrochemical initiation of polymerization.

#### 3.1.1. Polymer stability studies

**Fig. 2:**
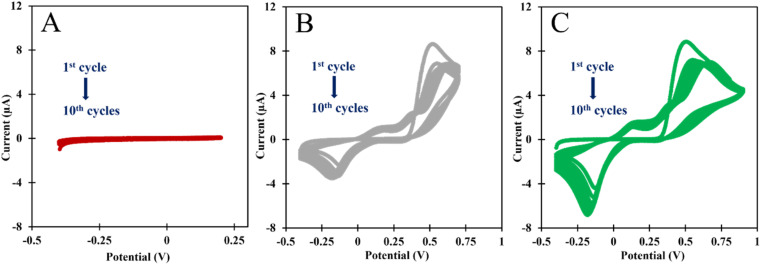
CVs of the ELP of the AMPTMA with different range potential. (A) −0.4 to +0.2 V; (B) −0.4 to +0.7 V; and (C) −0.4 to +0.9 V.

We started to evaluate the stability of the NIP/C-SPE material by changing the potential window or the potential range given by CV in the ELP process. The electrochemical parameters investigated for NIP/C-SPE polymerization were: 10 CV scans, a scan rate of 0.05 V s^−1^, and a potential window of (A) −0.4 to +0.2 V; (B) −0.4 to +0.7 V; and (C) −0.4 to +0.9 V (Fig. 2). No oxidation or reduction peak was observed in a potential window between −0.4 and +0.2 V, indicating that the system does not contribute enough energy in this potential range to generate a radical in the acrylamide group. When the C-SPE surface was exposed to a potential window with the monomer from −0.4 to +0.7 V, 3 oxidation peaks at +0.25, +0.32, and a prominent peak at +0.5 V and a reduction peak at −0.1 V were observed (Fig. 2B). Overall, the results show that an insulating film was generated as the peak current of the oxidation peaks decreases after each cycle.

**Fig. 3:**
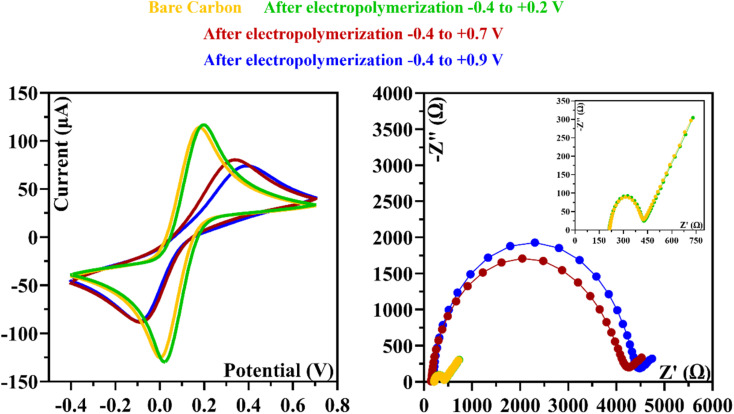
CV and EIS measurements were made after applying different potential ranges during the ELP of the monomer.

In the Fig. 2C, we analyzed the potential effect ranging from −0.4 to +0.9 V. The results are quite similar to those in Fig. 2B, and it appears that an insulating polymer has formed in the C-SPE surface. As for the stability of the sensor surface when the electrodes were exposed to a potential range between −0.4 and +0.7 V, a stable result was obtained in EIS and CV measurements after successive incubations with the buffer solution and measurements with the redox probe. The potential window of ELP selected for further studies was based on the values (*I*) and charge transfer resistance (*R*_CT_) of the analytical reaction and peak separation in CV measurements after successive incubations in PB buffer for 20 min. Since the polymer in the last condition exhibits higher isolation behavior, higher peak separation, and higher *R*_CT_ value, which hinders the electron charge exchange between the electrode surface and the solution, and consequently, could affect the sensitivity of the sensor (Fig. 3), we chose the potential range between −0.4 and +0.7 V for further studies.

#### 3.1.2. Imprinting stage

The selection of the polymer plays an important role in a successful cavity, which depends on several parameters, such as the degree of polymerization that depends on the parameters for ELP, pH, solvent, and monomer concentration. Poly-AMPTMA could be a promising polymer due is its positive charge, which has several advantages, including strong interaction with negatively charged targets, as in the case of glyphosate detection. Recently, Mashaalah Zarejousheghani described a MIP-based sensor using AMPTMA as a monomer for the selective detection of glyphosate^37^

**Fig. 4:**
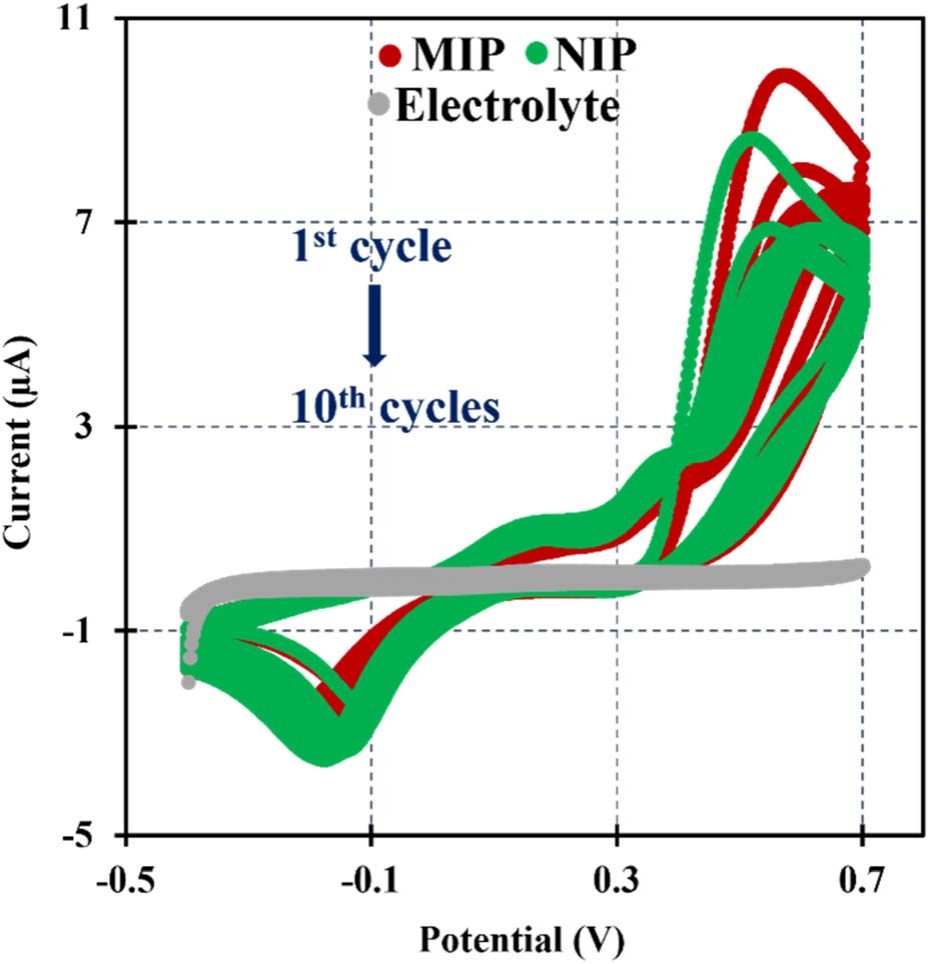
CVs of the ELP of the MIP and NIP spectra in PB buffer pH 5.8.

In this research the ELP of AMPTMA was produced by successive CV cycles on the pre-treated C-SPE surface. In the first cycle, the current increased towards the oxidation potentials and showed a peak current at about 0.5 and 0.6 V for NIP and MIP, respectively. This peak indicated the oxidation of AMPTMA and allowed the formation of the polymer (Fig. 4). The subsequent CV cycles showed a continuous decrease in the current of the system, confirming the growth of a non-conducting layer. When the electrode is exposed to a CV scan with buffer only, the peaks remain absent as expected.

#### 3.1.3. Electrochemical follow-up of imprinting stage

**Fig. 5:**
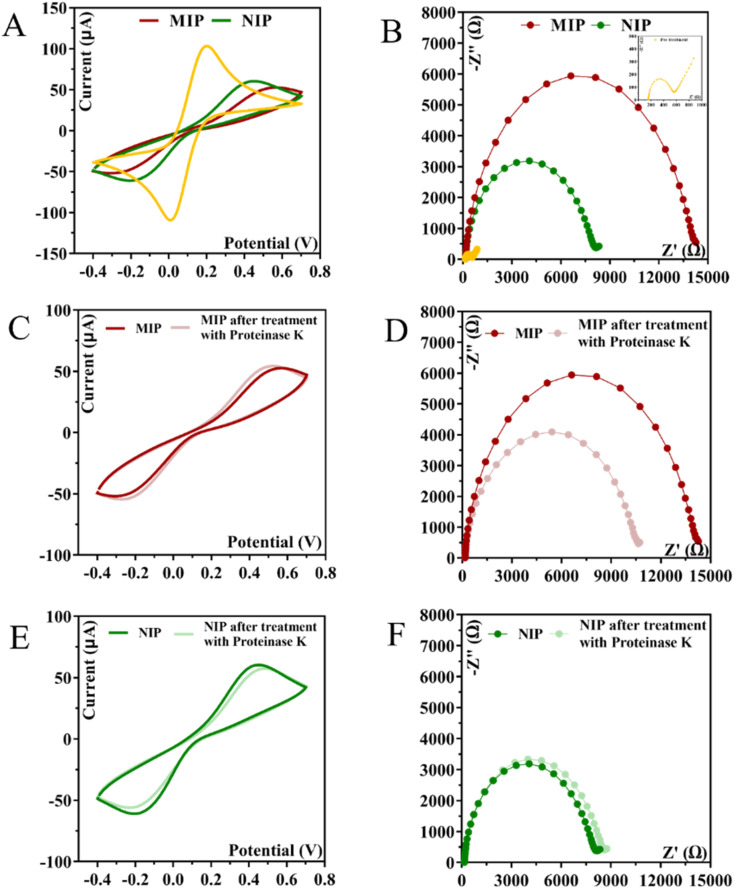
CV and EIS measurements were performed at various stages during the fabrication of MIP and NIP. Images (A) and (B) depict the after-polymerization process for MIP and NIP, respectively. The biosensor response after the removal of the target molecule is illustrated in (C) and (D) for MIP and (E) and (F) for NIP.

The CV profiles of the iron redox probe obtained for the C-SPE coated with the polymer film (MIP or NIP) on the WE confirm the presence of a highly blocked surface (Fig. 5A). During imprinting, the protein was mixed with the monomer and ELP was performed in PB buffer pH 5.8. The redox peaks of the iron redox probe on the clean C-SPEs decreased and the peak separation increased after the formation of the polymer layer. In parallel, the control film (NIP) was synthesized without the presence of CA 15-3 during ELP. The NIP polymer gave a lower *R*_CT_ compared to MIP (Fig. 5B), and this difference could only be attributed to the presence of protein in the MIP polymerization phase. Since CA 15-3 was located within the growing polymer, two related events occurred: The presence of CA 15-3 on the matrix changes the electrical properties of the surface on which the polymer grows compared to the monomer growing alone in NIP, which in turn affects polymer growth because the polymer is formed by an electrical stimulus acting on the electrode surface. In other words, the differences reflect the direct effect of the protein on the electrical properties of the surface and its indirect effect by promoting differential polymer growth through such different electrical characteristics.

CA 15-3 is removed from the polymer matrix to keep the cavities in shape when exposed to the proteinase K solution. The removal of the protein altered the typical electrical properties of the redox probe as this additional element was no longer present on the surface. After the removal of the template, the current in the CV spectra increased, indicating that the protein was removed from the polymer matrix.

The EIS measurements are consistent with the CV analysis. After ELP, a huge increase in *R*_CT_ was observed, which increased the diameter of the semicircle (Fig. 5D–F). For MIP material, the *R*_CT_ is higher than for NIP material, which is expected when the protein is present in MIP. The *R*_CT_ is different for MIPs and NIPs after exposure to proteinase K (Fig. 5D–F). When the NIP material was exposed to proteinase K, the *R*_CT_ value of the iron redox probe did not decrease, but only a slight change was observed. In contrast, the *R*_CT_ value of the MIP material decreased by 25% after exposure to proteinase K.

### 3.2. Physicochemical characterization of the surface modification

Analyses of morphological and chemical characteristics were carried out on (bio)mimetic materials and control films using scanning electron microscopy (SEM) and Raman spectroscopy.

#### 3.2.1. SEM analysis

**Fig. 6:**
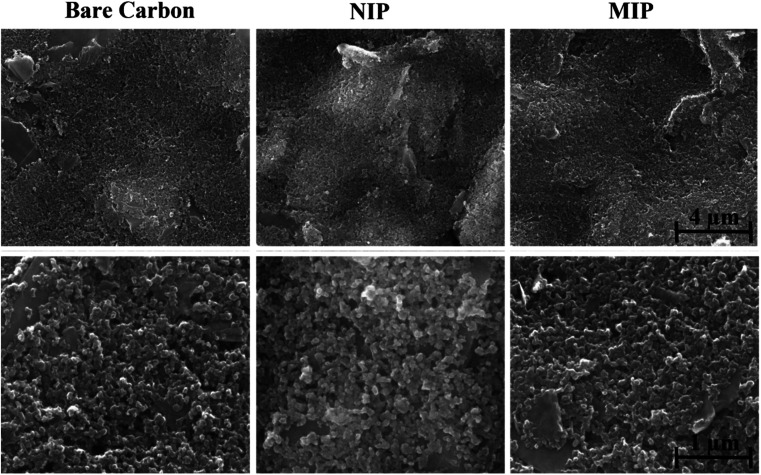
Results of SEM analysis of different stages of sensor construction.

The surface morphology of the carbon substrate and the substrate modified with MIP and NIP was examined by SEM (Fig. 6). The presence of imprinting sites could not be verified by SEM, as electron microscopy is not able to detect such small voids with sufficient resolution, so the two materials MIP and NIP appear to be similar. Nevertheless, Fig. 6 shows that the presence of the polymer on the C-SPE layer modified with MIP and NIP can be confirmed, as a thin film can be seen on the electrode surface.

#### 3.2.2. Raman spectroscopy analysis

**Fig. 7:**
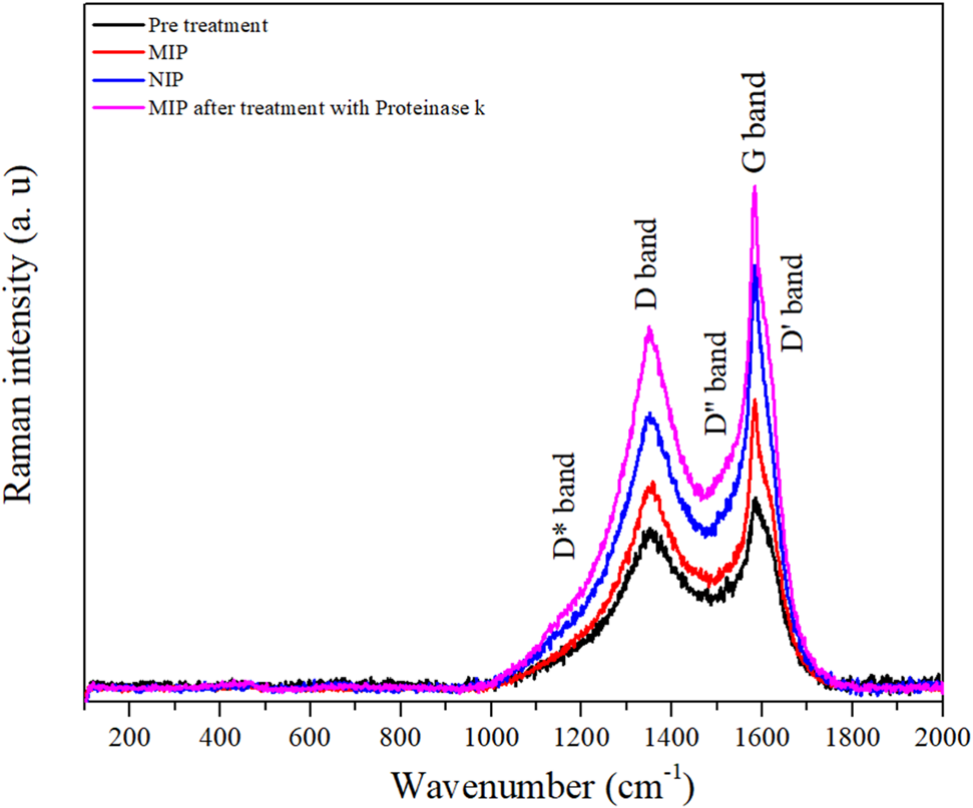
Raman spectra of the pre-treatment, MIP, NIP, and MIP after treatment with proteinase K.

In this study, the structural and chemical changes of the prepared (*i.e.* pretreatment, MIP, NIP, and MIP after treatment with proteinase K) were investigated by Raman spectroscopy. Raman spectroscopy was used to obtain information on the macroscale distribution of electrode modification of pretreatment, MIP, NIP, and MIP after treatment with proteinase K. The first-order Raman spectra show two main peaks between 1356 and 1586 cm^−1^, as shown in Fig. 7, which are characteristic features of graphitic carbons.^45^ The peak at around ∼1586 cm^−1^ is G band and is due to optical phonon mode with *E*_2g_ symmetry associated with an in-plane stretching of sp^2^ bonded carbon atoms.^46^

The peak at about 1356 cm^−1^ is referred to as the disorder-induced or D band and is associated with the *A*_1g_ symmetry breathing mode, which is associated with the oxidation of graphite and subsequent reduction of graphene oxide and significantly alters the basal plane structure of graphene.^47^

The degree of graphitization of a carbon material is generally characterized by the *I*_D_/*I*_G_ value in the Raman spectra, where *I*_G_ is the intensity of the G band corresponding to graphite and *I*_D_ is the intensity of the D band corresponding to defects and disorder in the graphene oxide. Table S1† shows the variation of the intensities and the relationship between the D and G bands. After a thorough analysis, it is important to highlight the increase in the variation of the *I*_D_/*I*_G_ ratio for the pre-treated (*I*_D_/*I*_G_ = 0.90), MIP (*I*_D_/*I*_G_ = 1.00), and NIP (*I*_D_/*I*_G_ = 0.86) samples, which suggests an increase in the number of defects in the carbon walls. These defects imply that there has been a modification in the working electrode with the polymerization process.^48^

It appears that the polymer has fewer structural defects than the carbon electrode. This seems surprising considering that graphite is a crystalline form of carbon, with a well-ordered hexagonal lattice structure. However, the electrodes are made of a commercial ink of unknown composition. If we compare the ratio of the NIP with the MIP, this material has fewer defects, which is to be expected since the MIP matrix contains the protein that can cause defects in the polymer. In addition, the *I*_D_/*I*_G_ ratio of the MIP sample decreased after treatment with proteinase K compared to other samples associated with protein extraction due to the presence of cavities in the polymer.

Overall, these results show that the surface of the carbon electrode was modified.

### 3.3. Analytical performance of the biosensor

#### 3.3.1. Calibration in buffer

The analytical performance of the biosensor was evaluated in PB buffer, pH 5.8. The SWV and current signals were measured by varying the CA 15-3 concentration within (0.001 and 10 U mL^−1^), as shown in Fig. S1.† The net current signals were inversely proportional to the CA 15-3 concentration. The calibration plot was constructed by plotting the current responses (*I*) against the logarithm of CA 15-3 concentration (Fig. S1†) Overall, the peak current at +0.4 V (shift to the right) decreased with increasing protein concentration. Under the optimized conditions, the MIP sensor showed a dynamic response range between 0.001 and 10 U mL^−1^.

Calibration of the MIP was expressed as current response (μA) = 0.0309 × [log(*C*), CA 15-3] + 0.1588 with a squared correlation coefficient of 0.9942 and a standard deviation on repeated testing 11%. Identical procedures were performed on non-imprinted biosensors (NIP) (Fig. S1†) which showed non-linear behavior. This indicates that within the concentration range studied, the response of the MIP is controlled by the interaction of CA 15-3 with the rebinding sites capable of discriminating the protein.

Furthermore, as far as we are aware, there are no studies in the literature on the detection of CA15-3 using a quaternary ammonium salt as a monomer. However, was reported in the literature some MIP-based sensors use other monomers. The results obtained emphasize the relevance of the comparison between the linearity range and the limit of detection (LOD) reported in the literature using MIP sensors for the determination of CA 15-3 and the results of this study, as can be seen in Table S2†. Compared to other methods, this work showed the best LOD, which allowed quantification of CA 15-3 down to 0.909 mU mL^−1^. This value is considerably below the threshold required for clinical assessment of breast cancer progression and recurrence, namely 30 U mL^−1^.

A possible explanation could be the fact that a monomer based on a quaternary ammonium salt, such as AMPTMA, can increase the sensitivity of an MIP sensor due to its unique properties. It forms strong and specific bonds with the target analytes due to the quaternary ammonium group, promotes electrostatic interactions, and increases reactivity during polymerization. This leads to greater efficiency in the formation of molecular recognition sites and to more stable and durable polymers that are ideal for analytical and biosensor applications.

#### 3.3.2. Calibration in serum samples

**Fig. 8:**
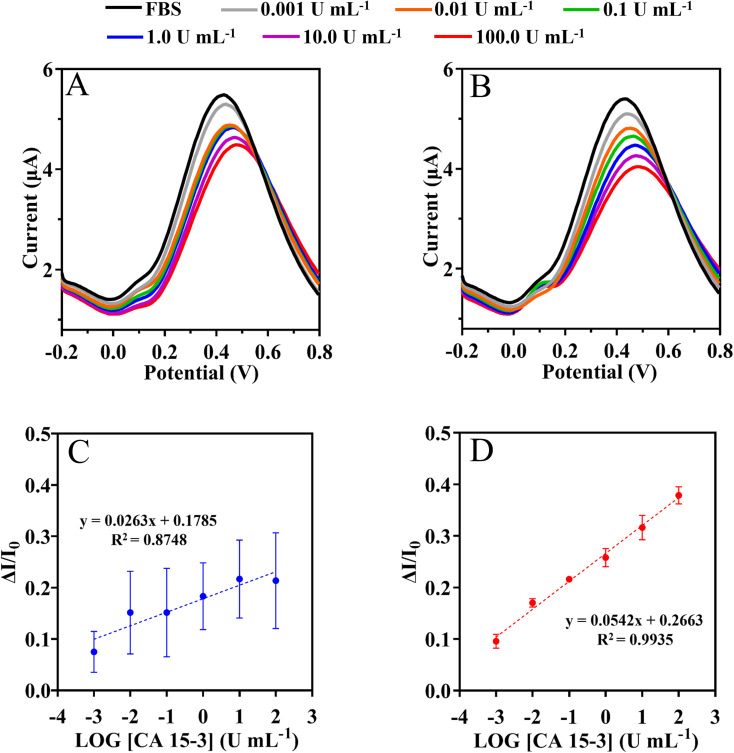
SWV measurements of NIPs (A) and MIPs (B) based biosensor and the corresponding calibration curves (C) and (D) respectively, in 5 mmol L^−1^ [Fe(CN)_6_]^3−^ and 5 mmol L^−1^ [Fe(CN)_6_]^4−^ by increasing CA 15-3 protein in the fetal bovine serum.

Because no access to real samples was possible, synthetic serum samples were used to assess the possible application of the device. For this purpose, instead of a real sample application, the calibration was made in a background of commercial serum and compared to that made previously in the buffer. The results obtained are presented in Fig. 8.

This assay was evaluated with serum 1000-fold diluted in PB buffer, pH 5.8. The SWV and current signals were measured by varying the CA 15-3 concentration within (0.001 and 100 U mL^−1^), as shown in Fig. 8. The calibration plot was constructed by plotting the current responses (*I*) against the logarithm of CA 15-3 concentration (Fig. 8). Overall, the peak current at +0.4 V (shift to the right) decreased with increasing protein concentration. Under the optimized conditions, the MIP sensor showed a dynamic response range between 0.001 and 100 U mL^−1^.

Calibration of the MIP was expressed as current response (μA) = 0.0542 × [log(*C*), CA 15-3] + 0.2663 with a squared correlation coefficient of 0.9935 and a standard deviation on repeated testing of 4%. Identical procedures were performed on non-imprinted material (NIP) (Fig. 8C), which showed non-linear behavior. Additionally, the NIP material had a lower slope (51% lower than MIP).

Overall, the LLRL of the calibration in buffer and serum is similar within 0.001 U mL^−1^ and the serum matrix appears to improve the slope by 43%. Overall, these results suggest that it is possible to successfully discriminate the detection of CA 15-3 in complex matrices.

#### 3.3.3. Selectivity studies

**Fig. 9:**
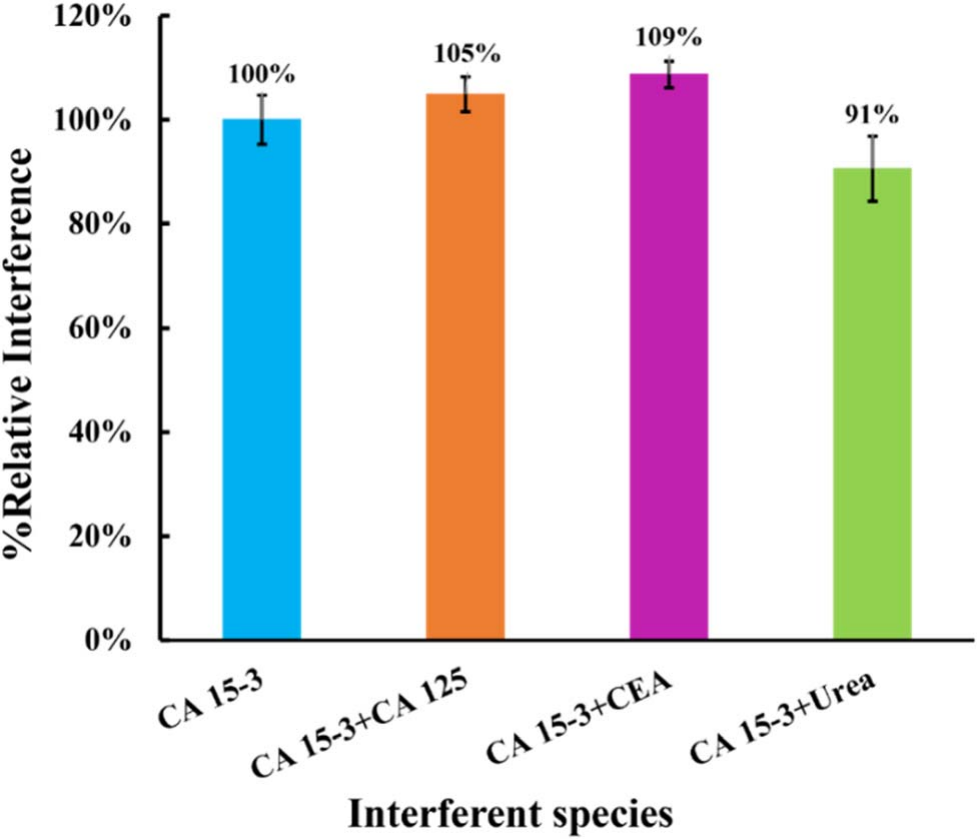
Selectivity studies using a competitive method between the target molecule and the interfering species. The interfering species studied were CEA, CA 125, and urea.

Selectivity is a crucial factor when evaluating the effectiveness of a biosensor under realistic conditions. In this study, the SWV response of solutions containing a potential interferent (diluted 1000-fold) and a concentration of CA 15-3 adjusted to 30 U mL^−1^ was compared to the response of a standard solution containing only CA 15-3 at the same concentration. Each solution was incubated on the sensor surface for approximately 20 min, the same duration used to calibrate the biosensor with CA 15-3 standard solutions. Components of the normal serum composition, namely CEA, CA 125, and urea, were chosen as interferents at concentrations of 2.5 ng mL^−1^, 35.0 U mL^−1^, and 0.2 mg mL^−1^, respectively. The signals obtained were compared and presented in Fig. 9. The relative values obtained with the interferents were compared to the percentage variation of the relative signal of isolated CA 15-3 protein.

This study showed that the binding of CEA protein (5%), CA 125 protein (9%), and urea (9%) was negligible when competing with the primary compound. These results indicate that the MIP surface has high selectivity, confirming the presence of imprinted cavities for CA 15-3. Consequently, these results suggest that these sensors have the potential to detect CA 15-3 in complex matrices.

## 4. Conclusions

This study delivered promising results that provide a solid foundation for significant advances in biomarker detection and monitoring, particularly in the context of breast cancer. The use of MIPs in combination with (bio)sensors has shown promise when it comes to the selective and sensitive detection of CA 15-3, a crucial biomarker in this disease. Research has also focused on the evaluation of quaternary ammonium cations as functional monomers for the development of imprinted polymers for protein detection, centered on the CA 15-3 biomarker for breast cancer. The integration of the MIP sensor into real-world applications, such as PoC testing or continuous monitoring of CA 15-3 levels in breast cancer patients, could significantly improve the health management of these patients. The ability to detect quickly and accurately important biomarkers such as CA 15-3 could enable earlier and more targeted intervention, which would have a positive impact on treatment outcomes and thus on patients' quality of life. Furthermore, the prospect of integrating these (bio)sensors into wearable or body-worn devices could revolutionize the way patients monitor their health, enabling an immediate response to fluctuations in biomarker levels and potentially reducing healthcare costs. Thus, this study not only contributes to the advancement of materials science and bioengineering, but also has the potential to make a tangible difference to patients' lives by providing an innovative approach to the diagnosis and monitoring of breast cancer and other health issues.

## Conflicts of interest

There are no conflicts to declare.

## Supplementary Material

RA-014-D4RA02051K-s001
