# Supplementary material for: An electrochemically synthesized molecularly imprinted polymer for highly selective detection of breast cancer biomarker CA 15-3: a promising point-of-care biosensor
Source: RSC Adv. 2024 May 13;14(22):15347–57. doi: 10.1039/d4ra02051k (PMC11089526; doi:10.1039/d4ra02051k)
Supplement: RA-014-D4RA02051K-s001 [file RA-014-D4RA02051K-s001.pdf]

Electronic Supplementary Material (ESI) for RSC Advances.
This journal is © The Royal Society of Chemistry 2024

Supporting Information

Electrochemically Synthesized Molecularly Imprinted Polymer for Highly Selective Detection of Breast Cancer Biomarker CA 15-3: A Promising Point-of-Care Biosensor

Table S1 – Spectral features of two-peak of the D and G band

Samples	D band	I_D	G band	I_G	I_D/I_G
Pre-treatment	1356	1496.88	1586	1657.60	0.90
MIP	1355	2259.15	1583	2266.07	1.00
NIP	1357	2940.55	1585	3420.36	0.86
MIP after treatment with proteinase k	1355	3819.17	1583	3703.42	1.03

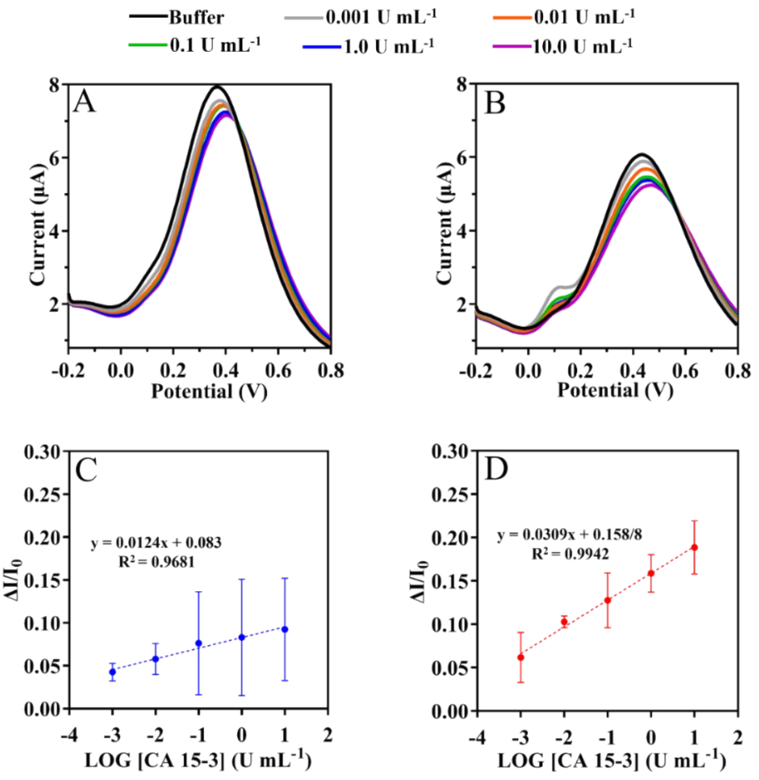

Figure S1– SWV measurements of the last stabilization in buffer and increasing standard concentrations of CA 15-3 in NIP (A) and MIP (B) prepared in PB buffer pH 5.8. Images C and D show the calibration curve of the normalized current response against the logarithmic concentration of CA 15-3 for NIP and MIP, respectively.

Table S2 – Analytical performance of sensor reported in the literature for the detection of CA 15-3.

**Sensing Approach**	**Transducer**	**Response Range U mL^{-1}**	**Limit of detection U mL^{-1}**	**Reference**
Sensor MIP	Electrochemical	0.25-10	0.05	[1]
Sensor MIP	Electrochemical	0.10-100	0.10	[2]
Sensor MIP	Electrochemical	5-50	1.50	[3]
Sensor MIP	Electrochemical	5-35	1.16	[4]
Sensor MIP	Electrochemical	0.001-10	0.000909	This Work

References

[1]. Gomes R.S., F.T.C. Moreira, R. Fernandes and M.G.F. Sales, PLOS ONE, 2018, **13**, 1-19.

[2]. Ribeiro J.A., C.M. Pereira, A.F. Silva and M.G.F. Sales, Biosensors and Bioelectronics, 2018, **109**, 246-254.

[3]. Pacheco J.G., M.S.V. Silva, M. Freitas, H.P.A. Nouws and C. Delerue-Matos, Sensors and Actuators B: Chemical, 2018, **256**, 905-912.

[4]. Oliveira, A. E. F, A. C. Pereira, L. F. Ferreira, Talanta,2023, **252**, 1-14.
